# Association of Neighborhood Measures of Social Determinants of Health With Breast, Cervical, and Colorectal Cancer Screening Rates in the US Midwest

**DOI:** 10.1001/jamanetworkopen.2020.0618

**Published:** 2020-03-09

**Authors:** Shaheen S. Kurani, Rozalina G. McCoy, Michelle A. Lampman, Chyke A. Doubeni, Lila J. Finney Rutten, Jonathan W. Inselman, Rachel E. Giblon, Kari S. Bunkers, Robert J. Stroebel, David Rushlow, Sagar S. Chawla, Nilay D. Shah

**Affiliations:** 1 Division of Health Care Policy and Research, Department of Health Sciences Research, Mayo Clinic, Rochester, Minnesota; 2 Robert D. and Patricia E. Kern Center for the Science of Health Care Delivery, Mayo Clinic, Rochester, Minnesota; 3 Division of Community Internal Medicine, Department of Medicine, Mayo Clinic, Rochester, Minnesota; 4 Department of Family Medicine, Mayo Clinic, Rochester, Minnesota; 5 Center for Health Equity and Community Engagement Research, Mayo Clinic, Rochester, Minnesota; 6 Department of Orthopaedics and Sports Medicine, University of Washington, Seattle

## Abstract

**Importance:**

Despite advances in cancer treatment and cancer-related outcomes, disparities in cancer mortality remain. Lower rates of cancer prevention screening and consequent delays in diagnosis may exacerbate these disparities. Better understanding of the association between area-level social determinants of health and cancer screening may be helpful to increase screening rates.

**Objective:**

To examine the association between area deprivation, rurality, and screening for breast, cervical, and colorectal cancer in patients from an integrated health care delivery system in 3 US Midwest states (Minnesota, Iowa, and Wisconsin).

**Design, Setting, and Participants:**

In this cross-sectional study of adults receiving primary care at 75 primary care practices in Minnesota, Iowa, and Wisconsin, rates of recommended breast, cervical, and colorectal cancer screening completion were ascertained using electronic health records between July 1, 2016, and June 30, 2017. The area deprivation index (ADI) is a composite measure of social determinants of health composed of 17 US Census indicators and was calculated for all census block groups in Minnesota, Iowa, and Wisconsin (11 230 census block groups). Rurality was defined at the zip code level. Using multivariable logistic regression, this study examined the association between the ADI, rurality, and completion of cancer screening after adjusting for age, Charlson Comorbidity Index, race, and sex (for colorectal cancer only).

**Main Outcomes and Measures:**

Completion of recommended breast, cervical, and colorectal cancer screening.

**Results:**

The study cohorts were composed of 78 302 patients eligible for breast cancer screening (mean [SD] age, 61.8 [7.1] years), 126 731 patients eligible for cervical cancer screening (mean [SD] age, 42.6 [13.2] years), and 145 550 patients eligible for colorectal cancer screening (mean [SD] age, 62.4 [7.0] years; 52.9% [77 048 of 145 550] female). The odds of completing recommended screening were decreased for individuals living in the most deprived (highest ADI) census block group quintile compared with the least deprived (lowest ADI) quintile: the odds ratios were 0.51 (95% CI, 0.46-0.57) for breast cancer, 0.58 (95% CI, 0.54-0.62) for cervical cancer, and 0.57 (95% CI, 0.53-0.61) for colorectal cancer. Individuals living in rural areas compared with urban areas also had lower rates of cancer screening: the odds ratios were 0.76 (95% CI, 0.72-0.79) for breast cancer, 0.81 (95% CI, 0.79-0.83) for cervical cancer, and 0.93 (95% CI, 0.91-0.96) for colorectal cancer.

**Conclusions and Relevance:**

Individuals living in areas of greater deprivation and rurality had lower rates of recommended cancer screening, signaling the need for effective intervention strategies that may include improved community partnerships and patient engagement to enhance access to screening in highest-risk populations.

## Key Points

**Question:**

How are area-level social determinants of health and rurality each associated with cancer screening practices?

**Findings:**

Among 78 302, 126 731, and 145 550 patients eligible for breast, cervical cancer, and colorectal cancer screening, respectively, in this cross-sectional study of adults receiving primary care in primary care practices in Minnesota, Iowa, and Wisconsin, census block group–level area deprivation and zip code–level rurality were separately associated with lower rates of screening in the 3 Midwest US states.

**Meaning:**

Individuals living in areas of greater deprivation and rurality have lower rates of cancer screening, underscoring the need for evidence-based interventions and targeted outreach to at-risk communities.

## Teaser

This cross-sectional study examines the association between area deprivation, rurality, and screening for breast, cervical, and colorectal cancer in patients from an integrated health care delivery system across 3 US Midwest states (Minnesota, Iowa, and Wisconsin).

## Introduction

One of every 3 persons is expected to be diagnosed as having cancer in his or her lifetime,^1,2^ with cancer ranking as the second leading cause of death in the United States.^3^ Although cancer-related mortality has declined over the past few decades, substantial racial/ethnic,^4,5^ rural,^4,6^ educational attainment,^5,7^ and socioeconomic^7,8^ disparities remain.^9^ Breast, cervical, and colorectal cancers are among the most frequently diagnosed cancers in the United States, and breast cancer and colorectal cancer are the second and fourth most prevalent causes of cancer-related deaths.^10,11^ The morbidity and mortality associated with these cancers can be reduced with timely guideline-recommended screening, diagnosis, and treatment.^12,13,14^ Therefore, lower rates of preventive screening for cancer may contribute to and exacerbate the disparities in cancer-related health outcomes in minority,^5,7^ rural,^6^ and low-income^7,8^ individuals.^8^ As such, it is important to improve our understanding of contemporary cancer screening disparities beyond the individual patient and potentially identify opportunities for population-focused and area-focused interventions.^6^

Addressing social determinants of health and ameliorating disparities in underserved populations are a priority, made increasingly more urgent and feasible as health systems shift toward value-based care models and a more holistic view of patients and population health.^15^ The social determinants of health framework hypothesizes that social and economic conditions shape population health, with the following 5 constructs associated with health outcomes: (1) economic stability, (2) educational level, (3) neighborhood and built environment, (4) health and health care, and (5) social and community context.^16^ However, prior studies examining disparities in cancer screening practices have not considered all aspects of this framework, instead focusing on select components that may not capture the full complexity of a patient’s situation.^15^ Yet, doing so is particularly important for preventive health behaviors, such as cancer screening, which involve access to care, adequate insurance coverage, health literacy, individual perceptions of health, and social capital.^9,15^

Given these complexities, public health and clinical interventions aimed at improving cancer screening rates and reducing cancer-related mortality would benefit from identification of areas with greatest gaps in care access and use. Geospatial disparities in social determinants of health are effectively captured by the area deprivation index (ADI), a validated composite area-based indicator composed of 17 US Census indicators spanning 4 domains, including poverty, educational level, housing, and employment,^17^ that is distinct from rurality. The ADI can be constructed for granular census-based regions such as the census block group, which contains 600 to 3000 persons,^18^ and denotes area-level socioeconomic disparities and disadvantages that cannot be measured or explained by traditional income, rurality, and race/ethnicity variables. Although cancer-related disparities based on income,^8^ rurality,^19^ and race/ethnicity^5^ have been individually described, it is not known how area-level deprivation alters recommended cancer screening practices independent of rurality. With 20% of the US population residing in rural areas,^20^ the present study examined rates of US Preventive Services Task Force (USPSTF)–recommended screening for breast, cervical, and colorectal cancer^21^ using patient-level data from 75 primary care practices in Minnesota, Iowa, and Wisconsin between July 1, 2016, and June 30, 2017. This work aims to increase understanding of the value of area-based metrics and their potential role in health care delivery and to identify opportunities for clinical and public health organizations to form partnerships to improve care.

## Methods

### Study Design

In this retrospective cross-sectional study, census block group–level cancer screening rates were analyzed for breast, cervical, and colorectal cancer among adults receiving primary care at 75 Mayo Clinic and Mayo Clinic Health System practices in Minnesota, Iowa, and Wisconsin. Census block groups are the smallest geographic unit for which the US Census Bureau publishes sample data.^22^ All data were abstracted and analyzed between December 2018 and September 2019. The study was approved by the Mayo Clinic Institutional Review Board and the requirement for informed consent was waived because the study was deemed minimal risk. This study followed the Strengthening the Reporting of Observational Studies in Epidemiology (STROBE) reporting guideline.

### Study Population

The present study included all persons empaneled to a Mayo Clinic or Mayo Clinic Health System primary care practice in Minnesota, Iowa, and Wisconsin who were eligible for breast, cervical, and/or colorectal cancer screening between July 1, 2016, and June 30, 2017. Screening eligibility was ascertained from electronic health records (EHRs) in accordance with USPSTF guidelines.^21^ The breast cancer cohort was composed of women aged 50 to 75 years and excluded women with a history of bilateral mastectomy.^21^ The cervical cancer cohort was composed of women aged 21 to 65 years and excluded women with a prior hysterectomy.^21^ The colorectal cancer cohort included adults aged 50 to 75 years and excluded patients with a prior total colectomy or previous colorectal cancer.^21^

The Census Geocoder, an address look-up tool that converts inputted addresses into latitude and longitude points,^23^ was used to link each patient address to a census block group. The coordinate points are accompanied by a match score (maximum value, 100), which details how closely the inputted address matches a candidate in the reference data^23^ (in this case, the 2010 US Census). Patients whose addresses could not be geocoded to a census block group with a match score greater than 60 or who were living in a zip code that did not have an associated rural-urban commuting area (RUCA) code were excluded.

### Primary Outcomes

Screening completion was ascertained from the EHRs in accordance with USPSTF guidelines.^21^ For breast cancer, screening completion was having 1 or more mammograms over a 2-year period.^21^ For cervical cancer, screening completion was undergoing cervical cytology in the past 3 years for women aged 21 to 65 years or cervical cytology and human papillomavirus co-testing in the past 5 years for women aged 30 to 65 years.^21^ For colorectal cancer, screening completion was having either colonoscopy within 10 years, flexible sigmoidoscopy or computed tomographic colonography within 5 years, multitarget stool DNA test within 3 years, or fecal occult blood test during the measurement year.^21^

### Explanatory Variables

Receipt of recommended cancer screening was examined as a function of ADI quintile. Census block group–level information necessary for ADI derivation was ascertained from 2012 to 2016 estimates of the 5-year American Community Survey (ACS).^24^ The ACS is an annual survey conducted by the US Census Bureau, which randomly samples housing units and provides population-level estimates representative of the noninstitutionalized US population.^24^ Seventeen census block group–level indicators were used, representing poverty, educational level, housing, and employment, to compute 2016 ADIs for all census block groups in Minnesota, Iowa, and Wisconsin (11 230 census block groups) (eTable in the Supplement). In-depth survey methods can be found on the US Census Bureau website.^24,25^

### Independent Variables

Rurality was assessed using patient zip code to identify corresponding RUCA codes, which were classified using published definitions for urban, rural, and highly rural areas.^26^ Zip code–level RUCA codes were used as individual risk factors to make patient-level inferences because census block group–level RUCA codes are not available through the US Department of Agriculture. Additional information regarding RUCA code descriptions can be found on the US Department of Agriculture website.^27^

Patient demographic characteristics (age, race/ethnicity, and sex) and comorbidities were ascertained from the EHRs. Race was classified as white or nonwhite per the EHRs. Race/ethnicity was assessed to investigate if there was an independent association between race and cancer screening completion after adjusting for other patient-level risk factors. The severity-weighted Charlson Comorbidity Index was calculated using codes from *International Classification of Diseases Ninth Revision* and *International Statistical Classification of Diseases and Related Health Problems, Tenth Revision (ICD-10) *for evaluation and management visits during the year before cohort entry.^28,29^

### ADI Derivation and Statistical Analysis

Modified 2016 ADIs were calculated based on the method described by Singh^17^ for all 11 230 census block groups in Minnesota, Iowa, and Wisconsin using 5-year ACS estimates.^24^ The variables used to derive ADIs are listed in the eTable in the Supplement. Variables were selected using a factor analysis approach,^17,30,31^ and missing values were substituted using single imputation. All variables were transformed to a rate per capita for the census block group. To improve on published ADI methods^17^ and prevent distortion of the ADI by larger continuous variables, such as income, these proportions were standardized to a mean (SD) of 0 (1), thereby ensuring that all variables in the modified ADI were scaled equally before weighting. Each variable was multiplied by its respective weight obtained from the factor score coefficient, and the 17 weighted measures were summed for each census block group to obtain the base score. Base scores were then standardized to a mean (SD) of 100 (20). The ADI was divided into quintiles for all analyses, with higher ADIs indicative of greater deprivation. A sensitivity analysis modeling the ADI as a continuous variable was also performed to ensure that findings remained consistent.

Multivariable logistic regression was used to identify the association between the ADI and cancer screening completion. Independent variables in the models included age, ADI quintile, rural status, Charlson Comorbidity Index, race, and sex (for the colorectal cancer outcome only).

Because of the small size of census block groups, traditional heat maps can be difficult to interpret. Therefore, a hot spot clustering analysis^32^ was performed to visualize area-level deprivation across the Midwest. Statistically significant hot spots represent concentration of census block groups with high deprivation; statistically significant cold spots represent concentration of census block groups with less deprivation. To be considered a statistically significant hot spot, a census block group will have a higher ADI (greater deprivation) and be surrounded by census block groups with high deprivation. This definition is similar for statistically significant cold spots but with lower ADIs (less deprivation). A census block group will result in a statistically significant *z* score if the local sum is very different from the expected local sum and the difference is too large to be attributable to random chance. Positive statistically significant *z* scores indicate more intense clustering of deprived census block groups (hot spots), and negative statistically significant *z* scores indicate intense clustering of less deprived census block groups (cold spots).

The testing was 2-sided, and the threshold of statistical significance for the study was *P* < .05. Analyses were conducted using SAS, version 9.4 (SAS Institute Inc) and Stata, version 15.1 (StataCorp LLC). A geographic information system map representing hot spot analysis of the ADI (Figure) at the census block group level was created in ArcMap, version 10.7 (Esri), using TIGER/Line Shapefiles from the US Census Bureau.

**Figure.**
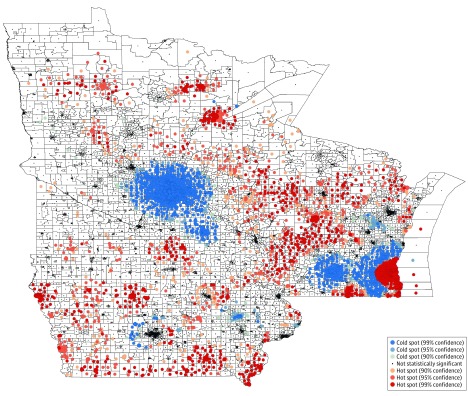
Census Block Group–Level Hot Spot Analysis of 2016 Area Deprivation Indexes Across Minnesota, Iowa, and Wisconsin Hot spots (red) indicate spatial clusters of census block groups with greater deprivation, and cold spots (blue) indicate spatial clusters of census block groups with less deprivation. White areas represent areas without statistically significant clustering. Higher area deprivation indexes indicate greater deprivation. In the key, “% confidence” means the statistical significance with a 99%, 95%, or 90% confidence level.

## Results

The study cohorts were composed of 78 302 patients eligible for breast cancer screening (mean [SD] age, 61.8 [7.1] years), 126 731 patients eligible for cervical cancer screening (mean [SD] age, 42.6 [13.2] years), and 145 550 patients eligible for colorectal cancer screening (mean [SD] age, 62.4 [7.0] years; 52.9% [77 048 of 145 550] women). More than 90% were of white race. Their baseline characteristics are summarized in Table 1. Distinct spatial clusters of census block group deprivation were identified throughout Minnesota, Iowa, and Wisconsin (Figure), with clustered cold spots (ie, areas of lower deprivation) concentrated around Minneapolis, Minnesota; Rochester, Minnesota; and Madison, Wisconsin.

**Table 1.** Patient Baseline Characteristics

Variable	Frequency, No. (%)		
	Breast Cancer (n = 78 302)	Cervical Cancer (n = 126 731)	Colorectal Cancer (n = 145 550)
Age, mean (SD), y	61.8 (7.1)	42.6 (13.2)	62.4 (7.0)
ADI quintile			
1	12 934 (16.5)	21 192 (16.7)	24 106 (16.6)
2	28 244 (36.1)	43 844 (34.6)	53 253 (36.6)
3	23 049 (29.4)	36 328 (28.7)	42 780 (29.4)
4	11 298 (14.4)	19 651 (15.5)	20 392 (14.0)
5	2777 (3.5)	5716 (4.5)	5019 (3.4)
Rural status			
Urban	27 948 (35.7)	49 889 (39.4)	51 100 (35.1)
Rural	48 779 (62.3)	74 866 (59.1)	91 556 (62.9)
Highly rural	1575 (2.0)	1976 (1.6)	2894 (2.0)
Charlson Comorbidity Index score			
1	49 646 (63.4)	102 423 (80.8)	89 349 (61.4)
2	14 026 (17.9)	15 906 (12.6)	26 761 (18.4)
3	7878 (10.1)	5251 (4.1)	15 024 (10.3)
≥4	3236 (4.1)	1651 (1.3)	6831 (4.7)
Race			
White	74 923 (95.7)	115 937 (91.5)	139 122 (95.6)
Nonwhite	3379 (4.3)	10 794 (8.5)	6428 (4.4)
Sex			
Female	NA	NA	77 048 (52.9)
Male	NA	NA	68 502 (47.1)

Abbreviations: ADI, area deprivation index (higher ADIs indicate greater deprivation); NA, not applicable.

### Association of Area-Level Deprivation With Cancer Screening

There was an inverse association between the ADI and screening rates for all 3 cancers. The adjusted probability of completing a screening decreased incrementally across ADI quintiles for the 3 cancers, as shown in the eFigure in the Supplement. The odds of completing recommended screening were decreased for individuals living in the most deprived (highest ADI) census block group quintile compared with the least deprived (lowest ADI) census block group quintile: the odds ratios (ORs) were 0.51 (95% CI, 0.46-0.57) for breast cancer, 0.58 (95% CI, 0.54-0.62) for cervical cancer, and 0.57 (95% CI, 0.53-0.61) for colorectal cancer (Table 2, Table 3, and Table 4).

**Table 2.** Factors Associated With Breast Cancer Screening Completion in Minnesota, Iowa, and Wisconsin

Variable	OR (95% CI)	*P* Value
Age, y		
50-64	1 [Reference]	NA
65-75	1.13 (1.09-1.18)	<.001
ADI quintile		
1	1 [Reference]	NA
2	0.81 (0.76-0.86)	<.001
3	0.72 (0.68-0.77)	<.001
4	0.66 (0.62-0.71)	<.001
5	0.51 (0.46-0.57)	<.001
Rural status		
Urban	1 [Reference]	NA
Rural	0.76 (0.72-0.79)	<.001
Highly rural	0.35 (0.32-0.39)	<.001
Charlson Comorbidity Index score		
0	1 [Reference]	NA
1	0.99 (0.94-1.04)	.56
2	1.00 (0.94-1.06)	.99
3	0.90 (0.82-0.98)	.02
≥4	0.81 (0.74-0.88)	<.001
Race		
White	1 [Reference]	NA
Nonwhite	0.48 (0.44-0.52)	<.001

Abbreviations: ADI, area deprivation index (higher ADIs indicate greater deprivation); NA, not applicable; OR, odds ratio.

**Table 3.** Factors Associated With Cervical Cancer Screening Completion in Minnesota, Iowa, and Wisconsin

Variable	OR (95% CI)	*P* Value
Age, y		
21-29	1 [Reference]	NA
30-49	1.86 (1.80-1.92)	<.001
50-65	1.21 (1.17-1.25)	<.001
ADI quintile		
1	1 [Reference]	NA
2	0.80 (0.77-0.83)	<.001
3	0.77 (0.74-0.80)	<.001
4	0.69 (0.66-0.72)	<.001
5	0.58 (0.54-0.62)	<.001
Rural status		
Urban	1 [Reference]	NA
Rural	0.81 (0.79-0.83)	<.001
Highly rural	0.78 (0.71-0.87)	<.001
Charlson Comorbidity Index score		
0	1 [Reference]	NA
1	1.00 (0.96-1.04)	.91
2	0.94 (0.88-1.00)	.04
3	0.80 (0.72-0.88)	<.001
≥4	0.86 (0.77-0.97)	.01
Race		
White	1 [Reference]	NA
Nonwhite	0.64 (0.61-0.67)	<.001

Abbreviations: ADI, area deprivation index (higher ADIs indicate greater deprivation); NA, not applicable; OR, odds ratio.

**Table 4.** Factors Associated With Colorectal Cancer Screening Completion in Minnesota, Iowa, and Wisconsin

Variable	OR (95% CI)	*P* Value
Age, y		
50-64	1 [Reference]	NA
65-74	1.41 (1.37-1.45)	<.001
≥75	1.22 (1.13-1.31)	<.001
ADI quintile		
1	1 [Reference]	NA
2	0.90 (0.87-0.94)	<.001
3	0.81 (0.78-0.85)	<.001
4	0.69 (0.66-0.73)	<.001
5	0.57 (0.53-0.61)	<.001
Rural status		
Urban	1 [Reference]	NA
Rural	0.93 (0.91-0.96)	<.001
Highly rural	0.94 (0.86-1.03)	.20
Charlson Comorbidity Index score		
0	1 [Reference]	NA
1	1.07 (1.04-1.11)	<.001
2	1.29 (1.23-1.35)	<.001
3	1.19 (1.11-1.26)	<.001
≥4	1.11 (1.04-1.17)	<.001
Race		
White	1 [Reference]	NA
Nonwhite	0.46 (0.44-0.49)	<.001
Sex		
Male	1 [Reference]	NA
Female	1.16 (1.13-1.19)	<.001

Abbreviations: ADI, area deprivation index (higher ADIs indicate greater deprivation); NA, not applicable; OR, odds ratio.

### Association of Rurality With Cancer Screening

Rurality had a separate association from the ADI in decreasing screening rates for breast, cervical, and colorectal cancer (Table 2, Table 3, and Table 4). Rurality was associated with breast cancer screening, with ORs of 0.35 (95% CI, 0.32-0.39) for patients living in highly rural areas and 0.76 (95% CI, 0.72-0.79) for patients living in rural areas compared with those living in urban areas. For cervical cancer screening, the ORs were 0.78 (95% CI, 0.71-0.87) and 0.81 (95% CI, 0.79-0.83) for highly rural and rural areas, respectively, compared with urban areas. For colorectal cancer screening, the ORs were 0.94 (95% CI, 0.86-1.03) and 0.93 (95% CI, 0.91-0.96) for highly rural and rural areas, respectively, compared with urban areas.

### Additional Patient-Level Factors Associated With Cancer Screening

Older patients were consistently more likely to complete cancer screening than younger patients (Table 2, Table 3, and Table 4). For breast cancer screening, the OR was 1.13 (95% CI, 1.09-1.18) for individuals aged 65 to 75 years compared with individuals aged 50 to 64 years. For cervical cancer screening, the ORs were 1.86 (95% CI, 1.80-1.92) for individuals aged 30 to 49 years and 1.21 (95% CI, 1.17-1.25) for individuals aged 50 to 65 years compared with individuals aged 21 to 29 years. For colorectal cancer screening, the ORs were 1.41 (95% CI, 1.35-1.45) for individuals aged 65 to 74 years and 1.22 (95% CI, 1.13-1.31) for individuals 75 years and older compared with individuals aged 50 to 64 years. Nonwhite patients were approximately half as likely to complete screening as white patients. For colorectal cancer, women were 16% more likely to complete the recommended screening than men. Finally, high comorbidity burden (Charlson Comorbidity Index score ≥4) was associated with increased odds of colorectal cancer screening (OR, 1.11; 95% CI, 1.04-1.17) but with decreased odds of breast cancer (OR, 0.81; 95% CI, 0.74-0.88) and cervical cancer (OR, 0.86; 95% CI, 0.77-0.97) screening (Table 2, Table 3, and Table 4).

## Discussion

Timely screening and early detection of breast, cervical, and colorectal cancer improve health outcomes,^33^ decrease cancer-related mortality,^33,34^ and reduce costs.^33,34,35,36^ Despite population improvements in cancer survivorship, statistically significant disparities remain. The present study built on earlier work that focused primarily on race/ethnicity–based^4,5^ and income-based^7,9^ disparities to identify a strong inverse association between area-level deprivation (as measured by the ADI), rurality, and USPSTF-recommended screening for breast, cervical, and colorectal cancer across Minnesota, Iowa, and Wisconsin. Individuals living in the 20% most deprived census block groups were almost half as likely to undergo recommended cancer screening as those living in the 20% least deprived census block groups, and individuals living in rural areas compared with urban areas were 7% to 24% less likely to complete a cancer screening. These associations were independent of one another and adjusted for patient age, comorbidity burden, and race. Overall, the results of the present study suggest that implementing area-based measures, such as the ADI, into practice and understanding differential cancer screening practices based on rural status may help inform and guide tailored interventions to meaningfully address disparities based on social determinants of health.^37^

Public health and policy interventions grounded in the environmental context may have enhanced effectiveness and practicality because of the reduced individual burden^38^ and consideration of the multifaceted nature of social determinants of health. In the case of cancer screening, community-based factors may intensify or supersede individual risk factors, including inadequate access to health care resources, poor community engagement with or distrust of the health care system, misperceptions about cancer screening, and other factors. Therefore, identifying disparities in cancer screening and other preventive health behaviors that stem from area-level deprivation and rurality signals the need for health care systems to form partnerships with local communities to enhance awareness, increase access, and improve overall health.

Issues potentially associated with accessing care were evident in this study, with deprivation altering cancer screening rates for the more frequent in-person tests and procedures, such as mammography (at least once every 2 years), compared with cervical cancer screening (every 3-5 years) or colorectal cancer screening (at least every 10 years for colonoscopy or stool-based testing that does not require in-person visits).^39,40^ Persons living in deprived and rural areas may especially benefit from comprehensive multilevel efforts,^37^ such as mobile screening service facilities or extended-hour screening clinics, which could enable patients in areas of high deprivation to seek care and mitigate access barriers faced by underserved populations, particularly in the rural Midwest.^19^

Contrary to national trends demonstrating similar screening completion rates for colorectal cancer among men and women,^41^ our study found that women were more likely to complete screening than men. This finding may be because of concurrent screening opportunities (with women also due for breast and cervical cancer screening) and more frequent contact with health care professionals because of pregnancy and childcare.^11,42^ Extensive media coverage, awareness, and promotion of women’s cancers may also contribute to higher rates of screening among women.^11^ Therefore, outreach efforts targeted specifically at men may be beneficial.

### Strengths and Limitations

To our knowledge, this is the first study to concurrently examine the association of area-level deprivation, as measured by the ADI, rurality, and race/ethnicity, with screening completion rates. The present study is strengthened by the high level of granularity in examining area deprivation, which renders these findings readily pertinent and actionable. Disparities in cancer screening in urban and rural settings across 3 Midwest states were examined, allowing investigation of the separate associations of deprivation and rurality, which has not been previously done to date. Focusing on data from 75 primary care practices within a single integrated health care delivery system allowed us to maximize data capture and accuracy of reported screening information. By identifying areas where patients are most likely to forego recommended cancer screenings, these findings can instruct health systems and practices that serve patients at highest risk. This study can further inform staffing decisions associated with care coordination, social work, and outreach.

This study has limitations. The findings are limited by the cross-sectional nature of this work, preventing the assessment of longitudinal associations between area-level deprivation and cancer screening rates and definitive ascertainment of causal relationships. Reliance on ACS data to compute the ADI makes our results vulnerable to nonresponse and imputation bias, although the main study outcomes (ie, cancer screening rates) were ascertained at the person level from the EHRs. The lack of racial/ethnic diversity is another limitation, with more than 90% of the cohorts composed of non-Hispanic white individuals. However, although not representative of the general US population, the cohorts are characteristic of the Midwest and particularly its more rural areas. We believe that this study is strengthened by its population because it allowed us to concurrently examine the associations of both the ADI and rurality across 3 Midwest states.

## Conclusions

The implications of this study extend beyond reporting of the association between area-level deprivation and cancer screening practices. Area-level deprivation likely alters all aspects of health care use and health behaviors and, as a result, should be considered in addition to rurality when developing interventions aimed at improving cancer screening rates. Further research is needed to examine how the ADI correlates with chronic disease management, receipt of other preventive services, and potentially preventable acute care use. Using the ADI has the potential to support and advance practice, public health, and policy by empowering health systems and community organizations to provide more patient-centered and equitable care.

## 

## Notes

Supplement. **eTable.** American Community Survey 5-Year Estimates and Factor Score Coefficients **eFigure.** Adjusted Percentage of Cancer Screening Completion Rates Among Primary Care Patients in Minnesota, Iowa, and Wisconsin
