# Supplementary material for: Association of Neighborhood Measures of Social Determinants of Health With Breast, Cervical, and Colorectal Cancer Screening Rates in the US Midwest
Source: JAMA Netw Open. 2020 Mar 9;3(3):e200618. doi: 10.1001/jamanetworkopen.2020.0618 (PMC7063513; doi:10.1001/jamanetworkopen.2020.0618)
Supplement: Supplement. — eTable. American Community Survey 5-Year Estimates and Factor Score Coefficients eFigure. Adjusted Percentage of Cancer Screening Completion Rates Among Primary Care Patients in Minnesota, Iowa, and Wisconsin [file jamanetwopen-3-e200618-s001.pdf]

Supplementary Online Content

Kurani SS, McCoy RG, Lampman MA, et al. Association of neighborhood measures of social determinants of health with breast, cervical, and colorectal cancer screening rates in the US Midwest. *JAMA Netw Open*. 2020;3(3):e200618. doi:10.1001/jamanetworkopen.2020.0618

eTable. American Community Survey 5-Year Estimates and Factor Score Coefficients

eFigure. Adjusted Percentage of Cancer Screening Completion Rates Among Primary Care Patients in Minnesota, Iowa, and Wisconsin

This supplementary material has been provided by the authors to give readers additional information about their work.

© 2020 Kurani SS et al. *JAMA Network Open*.

eTable. American Community Survey 5-Year Estimates and Factor Score Coefficients

US Census Indicator	2012-2016 ACS Table Reference, 5-year estimates	Factor Score Coefficient 2016
Median family income	B19013	-0.21740
Income disparity	B19001	0.05598
Families below poverty level	B17010	0.11151
% population below 150% poverty threshold	C17002	0.22992
Single parent household with dependents <18	B23008	0.04159
Households without a motor vehicle	B25044	0.04491
Households without a telephone	B25043	0.01567
Occupied housing units without complete plumbing	B25016	0.01140
Owner occupied housing units	B25003	-0.05758
Households with >1 person per room	B25014	0.01835
Median monthly mortgage	B25088	-0.10626
Median gross rent	B25064	-0.04952
Median home value	B25077	-0.08486
Employed persons ≥16 in white collar occupation	C24010	-0.05700
Civilian labor force unemployed (aged ≥16)	B23025	0.02541
Population aged ≥25 with <9yr education	B15003	0.06044
Population aged ≥25 with at least a high school education	B15003	-0.19303

© 2020 Kurani SS et al. *JAMA Network Open*.

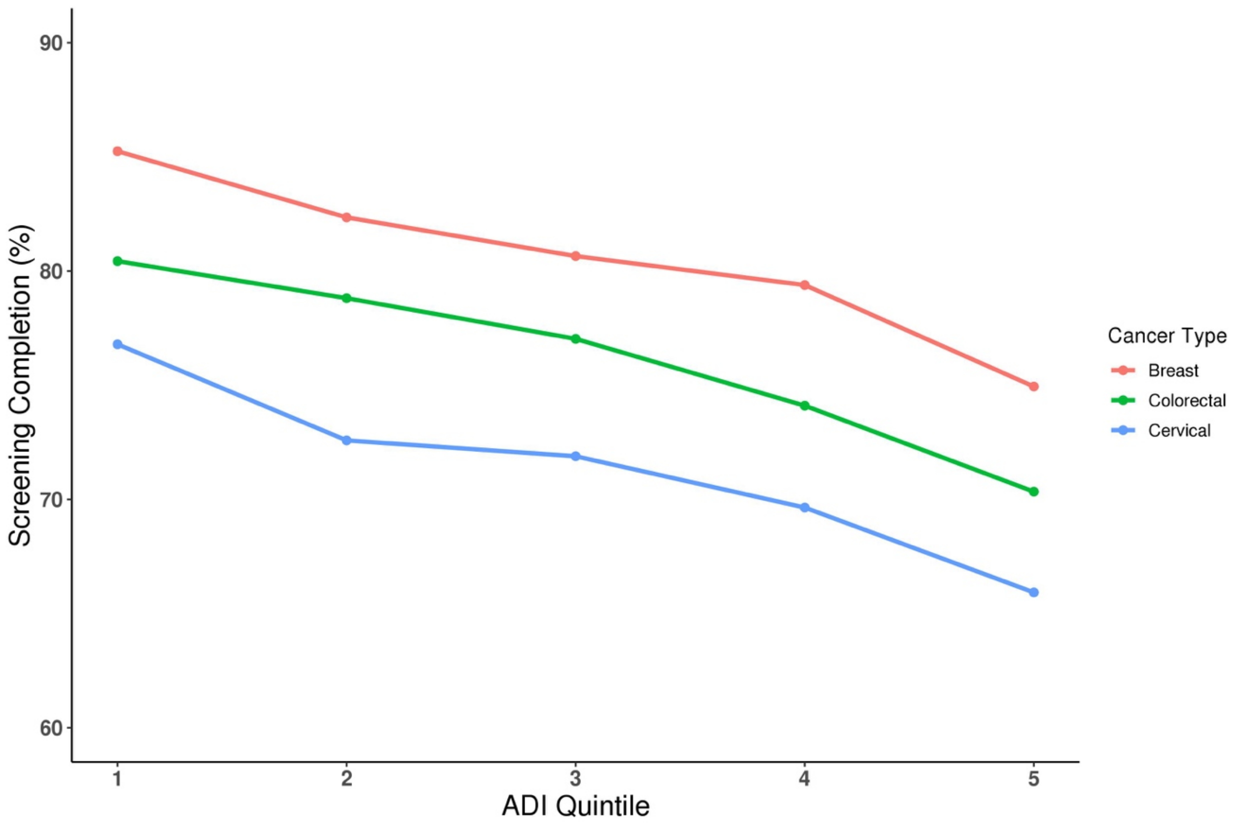

eFigure. Adjusted Percentage of Cancer Screening Completion Rates Among Primary Care Patients in Minnesota, Iowa, and Wisconsin

Adjusted for rural status, Charlson Comorbidity Index, race/ethnicity, sex (colorectal cancer only), and age.

© 2020 Kurani SS et al. *JAMA Network Open*.
